# Body Composition of Children with Type 1 Diabetes: A 20-Year Single-Center Comparative Study

**DOI:** 10.3390/children13070944

**Published:** 2026-07-18

**Authors:** Irina Halvadzhiyan, Stanimira Elkina, Chayka Petrova

**Affiliations:** Department of Pediatrics, Medical University—Pleven, 5800 Pleven, Bulgaria; st_elkina_bg@abv.bg (S.E.);

**Keywords:** body mass index, body composition, bioelectrical impedance analysis, children, type 1 diabetes, comparative study

## Abstract

**Highlights:**

**What are the main findings?**

- Compared with a cohort examined 20 years earlier, contemporary children with type 1 diabetes showed a trend of higher BMI values and increased fat-free mass, suggesting changes in body composition over time.
- Continuous glucose monitoring use was associated with lower HbA1c levels, lower insulin requirements, and lower BMI values compared with patients not using CGM.

**What are the implications of the main findings?**

- Sex-, age-, and diabetes duration-related differences in body composition should be considered when evaluating nutritional status and metabolic control in children with type 1 diabetes.
- Early implementation of diabetes technologies and individualized multidisciplinary care may contribute to better metabolic outcomes and a more favorable body composition profile during puberty and adolescence.

**Abstract:**

Background: Puberty in children with type 1 diabetes (T1D) is associated with increased insulin requirements, reduced insulin sensitivity, and changes in body composition. Objective: To evaluate body mass index (BMI) and body composition in children with T1D and compare the findings with data obtained 20 years earlier. Materials and Methods: This retrospective comparative study included two historical cohorts of children with type 1 diabetes (T1D) examined at the same tertiary diabetes center 20 years apart. A total of 293 participants were included: 145 age-matched children assessed in 2003 and 148 children assessed in 2023 (mean age 12.15 ± 4.42 years). Body mass index (BMI), fat mass (FM), and fat-free mass (FFM) were analyzed using bioelectrical impedance analysis, and the effects of insulin therapy, continuous glucose monitoring (CGM), physical activity, and family history of obesity were evaluated. Results: Mean BMI in 2023 was 19.85 ± 4.47 kg/m^2^; 85.8% of participants had normal body weight, 6.0% were overweight, and 8.1% had obesity. Children with a family history of obesity had higher BMI values than those without such history (23.2 ± 6.0 vs. 19.4 ± 4.3 kg/m^2^; *p* < 0.05). The highest BMI values in both sexes were observed in patients with T1D duration of 5–10 years. Compared with 2003, contemporary prepubertal boys had higher BMI values. Higher FM percentages in girls compared with boys were preserved. CGM use was associated with lower HbA1c, lower insulin dose requirements, and lower BMI values (all *p* < 0.05). Conclusions: Significant sex- and time-related differences in BMI and body composition were identified in children with T1D. CGM use was associated with better metabolic control and a more favorable body composition profile.

## 1. Introduction

In the presence of a persistent global trend of increasing prevalence of overweight and obesity in the pediatric population [1], insulin treatment in children with type 1 diabetes (T1D) may represent an additional risk factor for weight gain and body fat (BF) accumulation [2]. This has already been established in a number of studies and emphasizes the importance of body composition (BC) assessment for metabolic control and prevention of chronic complications of the disease [3,4]. In the long term, the incidence of cardiovascular events in individuals with T1D is higher compared with the general population when changes in BC and increased BF are observed, even in the absence of overweight and obesity [5]. Early intervention targeting modifiable risk factors, such as sedentary lifestyle, diabetes control, increased BF and dyslipidemia, has the potential to reduce this risk [6]. Bioelectrical impedance analysis (BIA) is a non-interventional BC assessment method widely used in clinical practice due to its speed, safety and ease of use in large groups of children [7]. Dual-energy X-ray absorptiometry (DEXA) is widely regarded as the gold standard for body composition assessment. However, its use in large population-based studies is limited. In addition, the method requires specialized software, which is not readily available in most tertiary care hospitals in Bulgaria.

To the best of our knowledge, no studies have analyzed generational differences in body composition in children with type 1 diabetes followed at the same clinical center over an extended period of time. Over the past two decades, significant changes have occurred in therapeutic technologies and strategies, including the widespread use of insulin pumps, as well as in glucose monitoring methods, with continuous glucose monitoring systems becoming increasingly common. In addition, the use of insulin analogues has almost completely replaced human insulin therapy.

The increasing prevalence of overweight and obesity among the pediatric population, along with the trend toward a lower age of onset of obesity, has also been observed in Bulgaria. Improved glycemic control has been suggested as a potential factor associated with increased fat accumulation. The underlying mechanisms include reduced glycosuria and enhanced glucose utilization, leading to a positive energy balance. These trends highlight the need for individualized therapeutic strategies, including optimization of insulin regimens, dietary management, and promotion of physical activity, in order to prevent long-term cardiometabolic complications.

The aim of the present study was to assess long-term trends in BMI, FM, and FFM in children with T1D over a 20-year period, and to investigate their association with glycemic control, physical activity, and family history of obesity in a contemporary cohort of children with T1D.

## 2. Materials and Methods

A total of 293 children and adolescents with T1D were included in the study: 148 assessed in 2023 (74 girls and 74 boys) and 145 children of similar age assessed in 2003. Children with newly diagnosed T1D within 2 months of diagnosis were excluded. In 2003, Petrova et al. conducted a study among 145 children with T1D and 148 healthy controls from Pleven, Bulgaria, in which BMI, BF percentage, BF (kg) and FFM were assessed [8]. At that time, all children with T1D were treated with human insulins in a basal-bolus regimen. No differences in BMI between children with T1D and the control groups were observed. Body fat percentage (BF%) was higher in girls with and without T1D compared with boys. A non-significant increase in BF percentage was observed in boys with T1D after the first year of the disease, while in girls fat accumulation was more pronounced during puberty. Fat-free mass (FFM) was in favor of boys, with no difference compared with the control groups, with the period of most intensive fat gain coinciding with pubertal development.

This is a retrospective comparative study based on the analysis of two temporally distinct cohorts of children and adolescents with T1D assessed at the same diabetes center, with a 20-year interval between cohorts. The 2023 cohort included children and adolescents with T1D followed up at the Diabetes Center in Pleven, with data collected in 2023. The results obtained were compared with similar data from a cohort of children and adolescents with T1D assessed at the same center in 2003. A control group of healthy children was not included in this study, as there were sufficient published data, including from recent years, that consistently described differences in BC between children with T1D and healthy controls [2]. Therefore, this analysis was focused on intergenerational differences in BC in children with T1D followed up at the same center under similar clinical conditions and by a similar investigator team.

In order to ensure a proper comparison between historical and current data on the metabolic status of children with T1D from our region, the subjects were divided into four identical groups according to disease duration, similarly to the methodology used in the previous study: up to 1 year, 1 to 5 years, 5 to 10 years and over 10 years from the onset of diabetes (Table 1).

To facilitate a clearer presentation of the study design and the comparisons performed between the different subgroups, the investigated parameters are summarized in Table 2.

As part of the medical history, information was collected on the presence of overweight, obesity, and type 2 diabetes (T2D) among first- and second-degree relatives. In addition, all participants were asked about their involvement in extracurricular physical activity. Information on physical activity was obtained during routine outpatient visits through a structured physician-administered interview with the child and their parents or legal guardians. Participants who reported regular participation in organized sports or recreational physical activity at least two to three times per week were classified as physically active. Physical activity was recorded as a dichotomous variable (yes/no) and included in the descriptive analysis.

Data on family history and physical activity were available only for participants included in the 2023 cohort. Body mass index (BMI, kg/m^2^), BF (percentage and kg), and FFM (kg) were analyzed. Body mass index (BMI) was assessed according to the Centers for Disease Control and Prevention (CDC) growth charts (www.cdc.gov/growthcharts), with normal weight defined as a BMI between the 5th and 85th percentile for age and sex. Overweight was defined as a BMI between the 85th and 95th percentile, and obesity as a BMI at or above the 95th percentile. The influence of the type of insulin therapy, the method of glycemic control monitoring, the level of physical activity and the family history of obesity on BMI and BC were assessed. Pubertal development was assessed using the Tanner scale. Height was measured with a Harpenden wall stadiometer to the nearest 0.1 cm and body weight was measured with a Tanita digital scale to the nearest 0.1 kg in lightly dressed and barefoot children. BMI was calculated using the standard formula: weight (kg)/height (m)^2^. Body composition was determined with a Tanita BC-543 Segmental Body Composition Monitor analyzer (Tanita Corporation, Tokyo, Japan), analyzing BF percentage, BF (kg) and FFM (kg). Glycated hemoglobin (HbA1c) was measured in whole blood by an immunoturbidometric method using a COBAS INTEGRA 400 device (Roche Diagnostics GmbH, Mannheim, Germany). The total daily insulin dose (TDD) was calculated based on the average daily insulin requirement reported by the patients and their parents and was expressed in units per kilogram of body weight (U/kg). The majority of children were treated with analogue insulins (90.5% vs. 9.5% with human insulins) in a basal-bolus regimen and at doses of 0.5–1.3 U/kg. Glycemic control was monitored using continuous glucose monitoring systems (50%) or a glucometer. Measurements were performed in a standard clinical setting during outpatient visits, with no fasting required, good hydration and without physical activity for at least 2 h before the assessment.

Written informed consent for participation and data processing was obtained from the parents/guardians of all participants included in the 2023 cohort. The study was conducted in accordance with the principles of the Declaration of Helsinki and approved by the Ethics Committee of Medical University—Pleven, Bulgaria (Protocol No. 7/29.04.2026).

### Statistical Analysis

Statistical analysis was performed using IBM SPSS Statistics, version 23.0 (IBM Corp., Armonk, NY, USA). Comparisons between the 2003 and 2023 cohorts were performed using the online MedCalc statistical calculator (MedCalc Software Ltd., Ostend, Belgium), available at https://www.medcalc.org// MedCalc Statistical SoftwareContinuous variables are presented as mean ± standard deviation (SD). Comparisons between independent groups were performed using the independent samples *t*-test, with Welch’s correction applied when equality of variances could not be assumed. A *p*-value < 0.05 was considered statistically significant.

## 3. Results

### 3.1. Characteristics of the 2023 T1D Cohort

**Figure 1:**
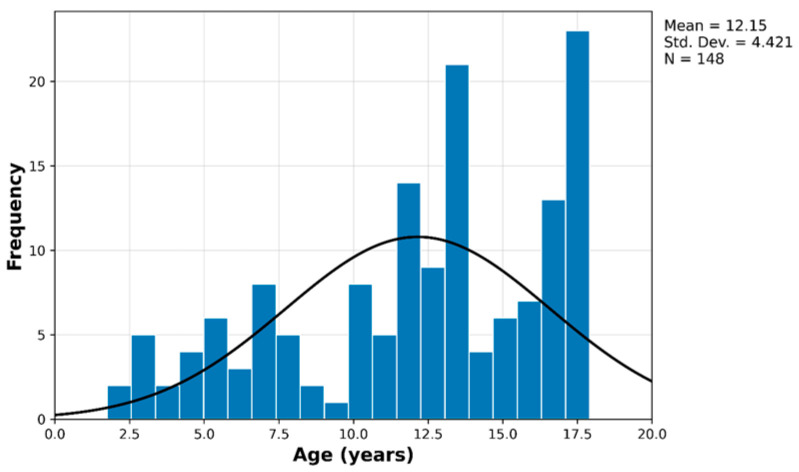
Age distribution of children and adolescents with T1D included in the 2023 cohort (*n* = 148).

All children followed at the Diabetes Center in 2023 were evaluated, with the exception of one newly diagnosed patient with disease duration below 2 months. The study population included 148 children with T1D (74 girls and 74 boys) with a mean age of 12.15 ± 4.42 years (Figure 1).

Normal body weight was observed in 127/148 children (85.8%), while overweight (BMI between the 85th and 95th percentile) was identified in 9/148 children (6.0%) and obesity (BMI ≥ 95th percentile) in 12/148 children (8.1%). Children with a family history of obesity demonstrated significantly higher BMI values compared with those without such history (23.2 ± 6.0 kg/m^2^ vs. 19.4 ± 4.3 kg/m^2^; *p* < 0.05). In addition, all children with overweight or obesity had parents with overweight and a family history of T2D. Only 20.3% of all children with T1D reported regular physical activity, most commonly football, cycling, and swimming. Regularly active participants were predominantly pubertal boys. Children without regular physical activity demonstrated a tendency toward higher BMI values, although without statistical significance (20.08 ± 5.1 kg/m^2^ vs. 19.8 ± 3.4 kg/m^2^).

### 3.2. BMI According to Sex and T1D Duration

To evaluate the influence of sex and disease duration on anthropometric characteristics, participants were analyzed according to T1D duration groups. BMI was significantly higher in boys from Group I (T1D duration ≤ 1 year; mean age 9.20 ± 4.98 years) compared with girls from the same group (*p* < 0.05) (Table 3).

The highest BMI values in both sexes were observed in Group III (T1D duration 5–10 years), corresponding to the period of active pubertal development (mean age 14.4 ± 3.07 years; Tanner stages 3–4).

### 3.3. Comparison Between the 2003 and 2023 Cohorts

To assess temporal trends in BMI over the past two decades, the contemporary cohort was compared with an age-matched cohort examined in 2003. Contemporary boys with T1D duration ≤ 1 year demonstrated significantly higher BMI values compared with boys from the same subgroup in 2003, whereas no significant differences were observed among girls (Table 3). Pubertal children of both sexes with diabetes duration between 5 and 10 years demonstrated significantly higher BMI values compared with the corresponding subgroups from the previous cohort, with the exception of boys with T1D duration > 10 years.

### 3.4. CGM Use and Metabolic Control

To evaluate the association between continuous glucose monitoring (CGM) and metabolic outcomes, participants were compared according to glucose monitoring method. By 2023, 50% of children with T1D followed at our center were using CGM systems (Table 4). Compared with patients performing self-monitoring with glucometers, CGM users demonstrated significantly lower HbA1c levels and lower total daily insulin dose requirements (*p* < 0.05). In addition to improved glycemic control, CGM users demonstrated significantly lower BMI values (19.03 ± 4.60 vs. 21.06 ± 4.82 kg/m^2^; *p* < 0.05). Children using CGM systems had a mean age of 10.4 ± 4.5 years, mean diabetes duration of 3.7 ± 2.9 years, and mean HbA1c of 8.0 ± 1.8%. In contrast, older patients with longer diabetes duration (5.9 ± 4.2 years) more frequently refused CGM use, which was associated with poorer glycemic control (HbA1c 10.1 ± 2.1%).

Because CGM users were significantly younger and had shorter diabetes duration than participants using glucometers, these findings should be interpreted as observed associations rather than evidence of a causal effect of CGM use.

### 3.5. Glycemic Control According to T1D Duration

To investigate the relationship between T1D duration and metabolic control, HbA1c levels and insulin requirements were analyzed across disease duration groups. With increasing T1D duration, glycemic control progressively deteriorated. Patients with T1D duration > 10 years demonstrated significantly higher HbA1c values compared with the remaining groups (HbA1c 10.32 ± 3.03%; *p* < 0.05) (Table 5). This subgroup consisted predominantly of adolescents with a mean age of 15.64 ± 1.97 years, who also required higher total daily insulin doses (1.10 ± 0.23 U/kg).

### 3.6. Body Composition Analysis

Body composition analysis demonstrated that prepubertal boys with T1D duration ≤ 1 year had higher fat mass percentages (FM%) compared with girls from the same subgroup. In all remaining subgroups, FM% was significantly higher in girls than in boys (*p* < 0.05) (Table 6 and Table 7). At the time of the current study, more than 95% of children with T1D were treated with insulin analogs, whereas all patients in the cohort examined 20 years earlier had received human insulin preparations. Although the sex-specific pattern of higher FM% in girls compared with boys was preserved, girls examined in 2023 generally showed lower FM% values than those examined in 2003. However, this difference reached statistical significance only in the subgroup with T1D duration > 10 years (30.9 ± 3.3% vs. 23.2 ± 5.6%; *p* = 0.003). These findings should be interpreted with caution because of the limited number of participants in this subgroup.

In contrast, fat-free mass was significantly higher in most groups of boys and girls examined in 2023, particularly among patients with diabetes duration between 1 and 10 years.

## 4. Discussion

This study aimed to evaluate changes in body composition (BC) in children with type 1 diabetes in Bulgaria in the context of global trends toward increasing body weight and BMI, and to assess the impact of modern therapeutic approaches, including insulin therapy and glucose monitoring, on these changes. The observed differences between the two cohorts may reflect the cumulative impact of advances in diabetes care and changes in lifestyle over the past two decades rather than the passage of time alone. Type 1 diabetes (T1D) is one of the most common chronic childhood diseases, the prevalence of which is increasing worldwide [9]. A trend toward increasing incidence has also been observed at our Diabetes Center over the past two decades. This observation is consistent with indirect demographic data indicating an increase in the relative proportion of children with type 1 diabetes despite a declining regional population (https://www.grao.bg/en, accessed on 17 May 2026).

The prolonged course of the disease with an onset as early as childhood is associated with the cumulative adverse impact of chronic hyperglycemia and insulin therapy on BC as well as with an increased risk of developing micro- and macrovascular complications. A number of publications report an increasing prevalence of overweight and adverse changes in BC in children with T1D, emphasizing the importance of its systematic assessment for the purpose of early prevention of the increased risk of vascular events in this population [2,10]. It is believed that the use of intensified insulin therapy to optimize glycemic control may be one of the factors contributing to the increased body fat (BF) accumulation [11]. On the other hand, a higher BF percentage is associated with an unfavorable cardiometabolic profile, including increased blood pressure, dyslipidemia, and elevated serum cholesterol levels [12], as well as slower muscle reoxygenation after physical activity in children with T1D, contributing to the development of insulin resistance [13]. The global trend towards an increase in overweight and obesity in childhood among those with T1D is also reported in countries with low economic status [14].

Body mass index (BMI) has long been used as a measure of body weight; however, it does not distinguish between BF and FFM. This represents a significant limitation in its interpretation, especially in the pediatric population [7]. The distribution of BC differs significantly depending on sex, with the increase in BMI with age in boys being mainly due to an increase in FFM, while in girls a predominant increase in BF is observed [15]. The dynamics of these changes are closely related to pubertal development and hormonal differences typical to this period [7,16,17].

Davis et al. [18] demonstrated that insulin deficiency at the diagnosis of T1D led to a catabolic, predominantly lipolytic state. Normalization of BC occurred approximately by the sixth week after the start of insulin treatment. Girls with T1D had a statistically significantly lower BF percentage both at diagnosis and at six weeks of therapy compared with both the control group of girls and the boys with T1D. Gender differences persisted one year after diagnosis as well. Despite the use of higher insulin doses, one year after diagnosis, girls demonstrated higher HbA1c values compared with boys [18]. The results of our study also similarly showed a lower BMI and BF percentage in prepubertal girls with newly diagnosed T1D compared with boys.

In recent years, the global trend of increasing prevalence of overweight and obesity among children aged 6 years has also been documented. In this regard, Semanova et al. presented results from the Feel4Diabetes project—a multicenter study conducted among children aged 6–10 years from six European countries (Belgium, Bulgaria, Finland, Greece, Spain, and Hungary), in which anthropometric parameters such as height, body weight and body mass index were assessed for the period of 2016–2018 [19]. According to the results presented, girls and boys in Bulgaria ranked third and fourth, respectively, in the prevalence of overweight and obesity among the European countries included. Even more concerning are the data from recent years, reflecting an unfavorable trend in the country. According to data from the National Center of Public Health and Analyses (NCPHA, https://ncpha.government.bg/, accessed on 17 May 2026) for 2023, every third child in Bulgaria at the age of 7 is overweight and every fifth one is obese, with boys prevailing for both parameters.

The increasing prevalence of obesity and the trend toward earlier onset are well established, with sex-specific differences in body composition dynamics [7,16]. Our findings indicate that prepubertal boys during the first year after the diagnosis of T1D have higher body weight, BMI, and BF percentage compared to age-matched girls, and these differences were observed in both study cohorts. One possible explanation is that this finding may be consistent with the reported increase in childhood overweight and obesity in Bulgaria; however, the present study was not designed to evaluate population trends directly or to determine whether these differences are independent of T1D or its treatment.

We found that the use of modern glucose monitoring technologies, particularly continuous glucose monitoring (CGM), was associated with better glycemic control, lower BMI and HbA1c levels, and reduced daily insulin requirements, consistent with findings from the literature [20,21,22,23]. In our center, CGM is predominantly used by children with shorter diabetes duration. These findings likely reflect stronger parental supervision in younger children, as well as the recent introduction of full reimbursement for glucose sensors during the final two years of the study period. However, these findings should be interpreted with caution, as CGM users differed significantly from participants using glucometers with respect to age and diabetes duration. Therefore, the observed differences cannot be attributed solely to CGM use but may also reflect differences in patient characteristics. The early onset of T1D and the use of higher insulin doses are believed to be among the factors contributing to the increased BF percentage in children with T1D [2]. The anabolic effect of insulin, expressed in the inhibition of lipolysis, stimulation of lipogenesis, increased cellular glucose uptake and reduction in energy losses by eliminating glycosuria, leads to a positive energy balance and may contribute to an increase in body weight and BF [24]. In addition, higher insulin doses may be associated with increased serum levels of leptin, which play a role in the regulation of energy balance and may contribute to increased body fat accumulation, especially in girls [25]. We observed an increase in BF percentage in girls during puberty as well as with the increase in disease duration. Literature data show that lifelong insulin resistance is more pronounced in girls compared with boys, which correlates with higher body fat percentage, with gender differences becoming more pronounced during puberty [18]. Despite a lower body weight at the early stages of the disease, girls exhibit higher insulin resistance, which correlates with data from studies in youth and adults demonstrating a more unfavorable metabolic profile and a higher incidence of diabetic complications in women with T1D [26].

Over a 20-year period, we observed a trend toward increasing BMI and FFM across all age groups in both girls and boys. These changes may be related to advances in diabetes care, including insulin therapy, as well as changes in dietary habits and other lifestyle factors; however, the present study was not designed to determine the relative contribution of these factors.

Paradoxically, despite higher BMI values in pubertal children with T1D, a lower BF percentage was observed compared to data from 2003. Although higher FFM values in 2023 may be interpreted as a marker of favorable physical development, in the context of progressively worsening glycemic control with increasing diabetes duration, the lower BF percentage should not be considered an indicator of a healthy body composition. Rather, one possible explanation is that it may reflect a relative catabolic state associated with suboptimal metabolic control; however, this hypothesis could not be verified in the present study.

The previously observed trend of higher body fat percentage in pubertal girls remains evident, although contemporary girls exhibit lower absolute FM values. The intergenerational differences in body composition may, at least in part, be related to changes in diabetes care over the past two decades, including the transition from human insulin preparations to insulin analogs; however, other therapeutic and lifestyle-related factors may also have contributed to these observations.

An additional contributing factor may be the high proportion of pubertal patients with suboptimal glycemic control (HbA1c > 7%), despite the use of modern therapeutic approaches and structured education. Despite advances in treatment, the observed deterioration of glycemic control with increasing diabetes duration remains a significant clinical challenge, as reflected by higher HbA1c levels in patients of both sexes with diabetes duration exceeding five years. Due to the lack of HbA1c data from the 2003 cohort, no conclusions can be drawn regarding temporal trends in glycemic control.

Some studies in children with T1D show an inverse correlation between HbA1c levels and BMI, in which poorer glycemic control is associated with a lower BMI, regardless of the use of higher insulin doses [27]. A similar relationship was found in the patients included in our study. During the period of active pubertal development, with longer duration of T1D and use of a higher total daily dose (TDD) of insulin, we observed a decrease in BMI and this trend was more pronounced in T1D boys at the end of puberty. At the same time, the highest HbA1c values were also recorded in this group. A mild catabolic state associated with suboptimal insulin action may explain the findings in our patients.

The observed tendency toward “adaptation” to suboptimal glycemic control in a substantial proportion of patients at our center highlights the need for additional strategies to improve metabolic control and reduce the risk of long-term complications of T1D.

A number of publications emphasize that socioeconomic status, ethnicity and lifestyle are key factors contributing to the epidemic of overweight and obesity as well as to the differences in body fat percentage in children and adults [3,12]. In this context, the higher BF percentage in children with T1D may be more closely related to an unbalanced diet and low physical activity than to the characteristics of insulin therapy [12]. Lower physical activity in children with T1D is often associated with fear of hypoglycemia after exercise, which is a barrier to active sports activity [28]. Data from the SWEET international multicenter project with 23,026 children with T1D included in this registry (2–18 years, T1D duration > 1 year) revealed a high percentage of overweight and obesity among children with T1D, 31.8% [29]. BMI SDS was higher in girls compared with boys, with a decrease in boys at the end of puberty. A high percentage of obesity was also reported in publications from the USA: overweight 22.9%, obesity 13.1%, higher weight among girls with T1D, 46.1% [30]. Predisposing factors were female sex, longer diabetes duration, higher TDD, and lower family income. In our Center, the prevalence of overweight and obesity is relatively low. However, all children with overweight and obesity in our cohort had parental overweight and a positive family history of T2D. These findings may reflect the combined influence of genetic predisposition, dietary habits, and family lifestyle, as well as suboptimal glycemic control.

In our cohort, an increase in BMI during puberty was observed in both sexes, with more pronounced changes in children with a family history of obesity and in those with low physical activity levels. In the present study, children with T1D who did not engage in regular physical activity tended to have higher BMI values, although the difference between physically active and inactive children did not reach statistical significance. Evidence from a systematic review indicates that reduced physical activity in children with T1D is associated with increased fat mass and a less favorable body composition [12]. Although physical activity in the present study was assessed using a simplified clinical classification, current ISPAD Clinical Practice Consensus guidelines recommend at least 60 min of moderate-to-vigorous physical activity daily, including muscle- and bone-strengthening activities at least three times per week [31]. Similarly, the 2026 ADA Standards of Care emphasize regular physical activity and healthy lifestyle interventions as essential components of comprehensive pediatric T1D management [32].

In this context, the promotion of regular physical activity should be a key component of clinical practice in our center. Although the present study was not designed to investigate pancreatic β-cell preservation or regeneration, the identification of age- and sex-related differences in body composition may contribute to more individualized management of children with T1D by supporting timely optimization of insulin therapy, dietary counseling, promotion of regular physical activity, and appropriate use of diabetes technologies.

Taken together, our findings, in conjunction with current ISPAD and ADA recommendations, are consistent with the conclusions of a recent 2026 systematic review [33], suggesting that routine assessment of body composition and early identification of excess adiposity should become integral components of pediatric T1D care. These findings are also consistent with recent evidence supporting the clinical value of routine body composition assessment beyond conventional anthropometric measures in adolescents with T1D [34].

Beyond glycemic control alone, regular assessment of body composition may facilitate individualized dietary counseling, optimization of insulin therapy, promotion of physical activity, and reduction in future cardiometabolic risk.

The present study has several limitations that should be considered when interpreting the results.

1. The relatively small number of patients in some subgroups, as well as the absence of a healthy control group, limits the ability to draw direct conclusions regarding population trends in body composition changes among Bulgarian children. In this context, the observed increase in fat-free mass in both sexes represents a novel finding that requires further clarification through prospective and controlled studies.
2. The retrospective design of the study does not allow for the establishment of causal relationships and limits control over data collection. In addition, because identical clinical variables were not available for both study cohorts, multivariable statistical analyses could not be performed in a reliable and comparable manner. Consequently, the independent contributions of age, sex, pubertal status, and diabetes duration to the observed differences in body composition could not be fully assessed, and residual confounding cannot be excluded. Future prospective studies with standardized collection of clinical variables and multivariable statistical analyses are warranted to better define the independent effects of these factors.
3. Possible differences in body composition assessment methods between the two study periods may have affected the comparability of the data. The comparison between the 2003 and 2023 cohorts covers a 20-year period during which substantial advances occurred in diabetes management, including the widespread use of insulin analogs, continuous glucose monitoring, structured diabetes education, and changes in lifestyle. Therefore, the observed differences between the two cohorts should not be attributed solely to the passage of time but rather interpreted as the combined influence of changes in diabetes care, treatment modalities, and lifestyle factors.
4. Detailed information regarding dietary habits and therapeutic modalities, including multiple daily injections and insulin pump therapy, was not systematically collected, which further limits interpretation of the findings. In addition, C-peptide concentrations were not routinely available for either study cohort and therefore could not be included in the present analysis. The relatively limited use of diabetes technologies should be considered, as only 50% of participants used continuous glucose monitoring systems, while insulin pump therapy was used by only a small proportion of patients. Furthermore, physical activity was assessed using a physician-administered interview rather than a validated questionnaire or objective measurement, which may have introduced reporting bias. Finally, the comparison between CGM users and participants using glucometers may have been influenced by differences in age and diabetes duration between the two groups, limiting causal interpretation of the observed associations.
5. An important limitation of the present study is the relatively poor glycemic control observed in a substantial proportion of patients, particularly in groups with longer diabetes duration, which may have influenced body composition and contributed to an underestimation or modification of the observed associations.

## 5. Conclusions

The present study identified significant sex- and time-related differences in body mass index and body composition in children and adolescents with type 1 diabetes over a 20-year period. Compared with the 2003 cohort, contemporary patients showed a trend toward higher BMI and increased fat-free mass, suggesting changes in body composition over time. The increase in BMI observed in prepubertal boys may be consistent with the increasing prevalence of childhood overweight and obesity reported in the general population, although the present study was not designed to evaluate population trends. Conversely, the lower body fat percentage observed in pubertal patients with longer disease duration and poorer glycemic control may be related to relative catabolic processes associated with suboptimal metabolic control, but this interpretation requires confirmation in future studies. Continuous glucose monitoring was associated with better glycemic control and a trend toward a more favorable body composition profile. These findings support an individualized and multidisciplinary approach to the care of children with T1D, with particular attention to body composition assessment and early intervention during puberty and adolescence.

## Figures and Tables

**Table 1:** Distribution of Patients According to Duration of Type 1 Diabetes (2023).

Group	Duration of T1D	Total, *n*	Boys, *n*	Girls, *n*
I	≤1 year	24	13	11
II	1–5 years	73	37	36
III	5–10 years	34	16	18
IV	>10 years	17	8	9

**Table 2:** Investigated Parameters and Comparative Groups in the Study.

Parameter	2003	2023	Sex-Based Comparison	Comparison According to T1D Duration	CGM Analysis
BMI	✓	✓	✓	✓	✓
Body fat (%)	✓	✓	✓	✓	–
Fat mass (kg)	✓	✓	✓	✓	–
Fat-free mass (kg)	✓	✓	✓	✓	–
HbA1c	–	✓	✓	✓	✓
Total daily insulin dose	–	✓	✓	✓	✓
CGM use	–	✓	–	✓	✓
Physical activity	–	✓	✓	✓	–
Family history of obesity	–	✓	✓	✓	–

Note: ✓—parameter analyzed; – data unavailable or analysis not performed. Abbreviations: BMI = body mass index; T1D = type 1 diabetes; CGM = continuous glucose monitoring.

**Table 3:** Comparison of BMI between 2003 and 2023 in Children with T1D According to Sex and Disease Duration.

Group/Sex	*n* (2003)	BMI (kg/m^2^), 2003	*n* (2023)	BMI (kg/m^2^), 2023	*p*-Value
I (≤1 year), boys	9	15.9 ± 1.6	13	19.5 ± 4.6	0.0365
I (≤1 year), girls	17	15.8 ± 2.6	11	15.4 ± 1.4	NS
II (1–5 years), boys	32	19.5 ± 3.2	37	19.8 ± 4.0	NS
II (1–5 years), girls	36	17.9 ± 2.5	36	19.9 ± 6.0	NS
III (5–10 years), boys	19	20.0 ± 2.7	16	22.9 ± 3.4	0.0082
III (5–10 years), girls	23	18.9 ± 2.5	18	21.1 ± 4.3	0.0467
IV (>10 years), boys	10	21.4 ± 3.0	8	19.4 ± 4.2	NS
IV (>10 years), girls	2	19.8 ± 0.8	9	21.8 ± 5.3	NS

Data are presented as mean ± standard deviation (SD). BMI = body mass index; NS = not statistically significant (*p* > 0.05). *p*-values represent comparisons between 2003 and 2023.

**Table 4:** Demographic and Clinical Characteristics of Participants According to CGM Use.

Variable	CGM Users (*n* = 74)	Non-CGM Users (*n* = 74)	*p*-Value	Total (*n* = 148)
Age, years	10.39 ± 4.55	13.91 ± 3.52	<0.001	12.15 ± 4.42
Diabetes duration, years	3.70 ± 2.95	5.97 ± 4.25	<0.001	4.83 ± 3.82
Total daily insulin dose, U/kg/day	0.89 ± 0.25	1.04 ± 0.25	0.001	0.97 ± 0.26
HbA1c, %	8.02 ± 1.77	10.07 ± 2.12	<0.001	9.05 ± 2.20

Data are presented as mean ± standard deviation (SD). CGM = continuous glucose monitoring; HbA1c = glycated hemoglobin; U/kg/day = units per kilogram per day. *p*-values were calculated using the independent samples *t*-test.

**Table 5:** Age, Total Daily Insulin Dose, and HbA1c Levels According to Diabetes Duration.

Group	Age (Years)	Total Daily Dose (U/kg/Day)	HbA1c (%)
I	9.20 ± 4.98	0.76 ± 0.24	8.40 ± 1.52
II	11.26 ± 4.21	0.94 ± 0.24	8.86 ± 2.10
III	14.40 ± 3.07	1.07 ± 0.25	9.25 ± 2.00
IV	15.64 ± 1.97	1.10 ± 0.23	10.32 ± 3.03

Data are presented as mean ± standard deviation (SD). HbA1c = glycated hemoglobin; U/kg/day = units per kilogram per day.

**Table 6:** Body Composition Parameters in Children with T1D Examined in 2003 and 2023 According to Disease Duration and Sex.

Group/Sex	Year	*n*	BMI (kg/m^2^)	FM (%)	FM (kg)	FFM (kg)
I (≤1 year) girls	2003	9	15.9 ± 1.6	21.1 ± 5.7	7.1 ± 3.1	30.0 ± 4.8
	2023	11	15.4 ± 1.4	16.6 ± 2.4	6.2 ± 1.7	31.2 ± 7.6
I (≤1 year) boys	2003	17	15.8 ± 2.6	15.1 ± 5.2	4.8 ± 3.4	25.4 ± 6.2
	2023	13	19.5 ± 4.6	18.7 ± 4.7	7.6 ± 1.7	32.1 ± 13.3
II (1–5 years) girls	2003	32	19.5 ± 3.2	25.5 ± 5.5	11.9 ± 2.9	31.7 ± 6.2
	2023	36	19.9 ± 6.0	23.1 ± 5.6	13.1 ± 7.4	40.2 ± 12.4
II (1–5 years) boys	2003	36	17.9 ± 2.5	18.2 ± 4.1	8.0 ± 3.8	35.2 ± 6.1
	2023	37	19.8 ± 4.0	16.9 ± 5.1	9.2 ± 5.4	42.4 ± 14.9
III (5–10 years) girls	2003	19	20.0 ± 2.7	28.1 ± 5.8	13.1 ± 3.2	31.3 ± 4.6
	2023	18	21.1 ± 4.3	22.1 ± 5.5	11.6 ± 6.3	38.0 ± 10.8
III (5–10 years) boys	2003	23	18.9 ± 2.5	19.4 ± 3.0	9.1 ± 2.1	37.0 ± 6.1
	2023	16	22.9 ± 3.4	17.6 ± 5.7	11.1 ± 4.7	48.7 ± 10.7
IV (>10 years) girls	2003	10	21.4 ± 3.0	30.9 ± 3.3	15.7 ± 2.4	34.3 ± 8.6
	2023	9	21.8 ± 5.3	23.2 ± 5.6	12.2 ± 5.7	38.4 ± 6.9
IV (>10 years) boys	2003	2	19.8 ± 0.8	18.0 ± 1.4	8.9 ± 1.2	46.6 ± 7.2
	2023	8	19.4 ± 4.2	15.5 ± 5.9	8.8 ± 5.8	44.5 ± 11.7

Abbreviations: BMI = body mass index; FM = fat mass; FFM = fat-free mass.

**Table 7:** *p*-Values for the Comparison of Body Composition Parameters between 2003 and 2023 According to Sex and Disease Duration.

Group/Sex	BMI *p*	Body Fat (%) *p*	Fat Mass (kg) *p*	Fat-Free Mass (kg) *p*
I girls	0.473	0.051	0.451	0.673
I boys	**0.018**	0.057	**0.007**	0.112
II girls	0.729	0.080	0.374	**0.008**
II boys	**0.018**	0.234	0.275	**0.009**
III girls	0.362	0.115	0.374	**0.023**
III boys	**0.001**	0.613	0.127	**0.001**
IV girls	0.845	**0.003**	0.116	0.266
IV boys	0.808	0.311	0.965	0.773

*p*-values were calculated using the independent samples *t*-test (Welch correction). Bold values indicate statistical significance (*p* < 0.05).

## Data Availability

The data presented in this study are available from the corresponding author upon reasonable request. The data are not publicly available due to privacy and ethical restrictions and are stored at the Diabetes Center, University Hospital “Dr. Georgi Stranski”, Pleven, Bulgaria.

## Abbreviations

The following abbreviations are used in this manuscript:

BC	Body composition
BMI	Body mass index
BIA	Bioelectrical impedance analysis
T1D	Type 1 diabetes
FM	Fat mass
FFM	Fat-free mass
